# Subcutaneous Sacro Coccygeal Myxopapillary Ependymoma: A Case Report and a Comprehensive Review of the Literature Reappraising Its Current Diagnostic Approach and Management

**DOI:** 10.7759/cureus.14931

**Published:** 2021-05-10

**Authors:** Subramaniam Ramkumar, Cliff A Wanniang, Anju Risa Wahlang, J C Alepes Lamin

**Affiliations:** 1 Pathology, Woodland Hospital, Shillong, IND; 2 Surgery, Civil Hospital Shillong, Shillong, IND; 3 Radiology, Woodland Hospital, Shillong, IND; 4 Radiation Oncology, Civil hospital Shillong, Shillong, IND

**Keywords:** myxopapillary ependymomas, subcutaneous, extra spinal ependymomas, sacro coccygeal swellings, ependymal cell rests, mucinous tumours, sacrum

## Abstract

Sacrococcygeal myxopapillary ependymoma (MPE) is an uncommon type I glial tumor detected most frequently in the lumbosacral area of adolescents and children. It is usually presented as an intradural ependymal tumor that originates from the filum terminale and other locations within the ventricular system along the craniospinal axis. In rare cases, however, MPE may develop as a primary subcutaneous tumor in the sacrococcygeal area. Tumors can also appear as a dorsal sacrococcygeal growth or subcutaneous nodule. In this case report, we describe a rare case presenting as a subcutaneous sacrococcygeal mass in an elderly female that was subsequently resected and confirmed as subcutaneous MPE. The current standard treatment for MPE is maximal surgical resection with or without postoperative radiotherapy based on the locoregional extent and histological grading. However, there is limited evidence that radiotherapy for oligometastatic foci improves longevity or extends the time to recurrence. In addition to this case report, we provide a comprehensive review of similar cases and case series in the medical literature. Prospective studies evaluating the efficacy of resection and/or radiotherapy are required for improved management of extradural MPE.

## Introduction

Background

Ependymomas are slowly growing glial cancers of the central nervous system (CNS) [1]. While these lesions account for 60% of glial tumors originating in the spine, the overall incidence is extremely low. Recent studies have proposed that these neoplasms arise from radial glia, cells that act as scaffolds for cell migration during CNS development and then normally differentiate into neurons and other mature glial cells [2]. Myxopapillary variants of ependymomas are also rare. These myxopapillary ependymomas (MPEs) are well-circumscribed, slow-growing, grade II tumors that can appear throughout life but arise primarily in younger adults, with an average age of 36 years at the time of diagnosis [3]. They are distinguished from lower grade 2 ependymomas and malignant grade 3 ependymomas by differences in growth rate and site of origin, which in the case of MPE is most frequently in the caudal spinal cord close to the filum terminale, conus medullaris, and cauda equina. However, MPEs may also arise in the intraventricular space, brain parenchyma, and cervical thoracic spine [3]. If intraventricular, these tumors can sometimes bleed into the cerebrospinal fluid, causing a limited spread to other regions inside the CNS [3].

Extraspinal ependymomas arising in the sacrococcygeal region are also observed, as either presacral or postsacral masses. While rare, these show a stronger propensity to spread systemically through lymphovascular dissemination [2,4,5]. Presacral masses can exert pressure on the bowel or bladder, while postsacral masses can present as intergluteal swellings [2,5,6]. The differential diagnoses for these sacrococcygeal masses also include pilonidal sinus, neurogenic tumor, sacrococcygeal teratoma, soft tissue sarcoma, and metastatic carcinoma [3]. The preferred imaging modality for detecting, grading, and staging these lesions is magnetic resonance imaging (MRI). Leptomeningeal distribution along the spinal axis can also be detected using MRI [7].

The grade and location of an ependymoma determine its management. Metastasis occurs more frequently (18%) in the very uncommon posterior extraspinal ependymomas, but rarely in the anterior extrathecal (presacral) ependymomas (7%). Metastatic sacrococcygeal MPE deposits have been observed in regional lymph nodes, liver, lungs, and bone [8]. Due to this proclivity for systemic spread, the current treatment standard for spinal cord ependymomas most frequently encountered in adults is maximum surgical resection along with postoperative radiotherapy, especially in cases of partial resection [3,9-11]. Here, we describe a rare case of extradural sacrococcygeal subcutaneous ependymoma in an elderly female. Due to its rarity, there is a paucity of information for determining the best pathological diagnostic approach and for ruling out the closest differential diagnoses. This article describes in detail the cytomorphologic, histomorphologic, and immunohistochemical features of MPEs, the closest differential diagnoses (with emphasis on radiologic diagnosis), and current clinical management.

## Case presentation

Case report

**Figure 1:**
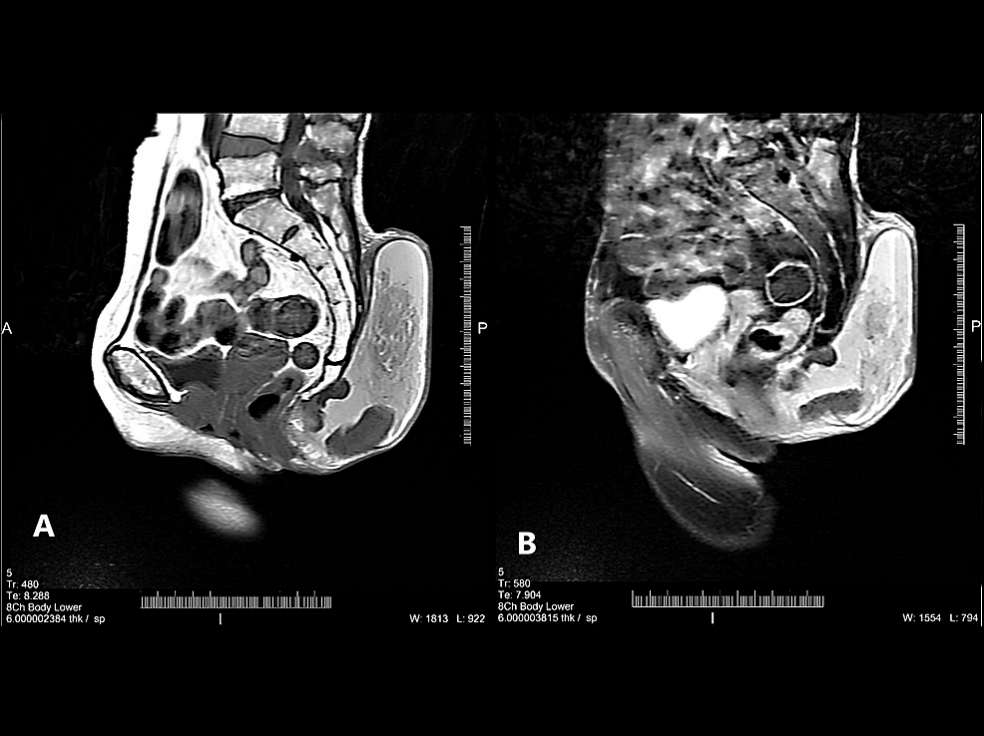
Subcutaneous sacrococcygeal ependymoma CT sagittal view (CT plain and CT contrast). (A) A large well-defined lesion is seen involving the sacrococcygeal region in the skin subcutaneous plane. Anteriorly, the lesion abutting coccyx vertebral body with no bony erosion seen (image plane sagittal T1 study). (B) Heterogenous enhancement on post-contrast images with multiple non-enhancing areas. The lesion shows the surrounding T2W1 hypointense capsule.

A 69-year-old female presented with a history of swelling over the sacrococcygeal region for the past one and a half years. The swelling was initially small but gradually increased in size, at presentation reaching 15 × 15 cm^2^. On examination, the swelling was soft and had a baggy feeling on palpation. Despite its size, the mass was painless and associated with no neurosensory deficits. CT scan revealed a large, well-defined, hyperintense lesion around 13 × 10 cm^2^ in size over the sacrococcygeal region in the subcutaneous plane. The anterior aspect abutted the sacrum, coccyx, vertebral bone but without invasion, while the inferior aspect compressed the bilateral ischiorectal fossa fat pad. There was no extension into the anal canal. The lesion had a heterogeneous enhancement on post-contrast images but with multiple non-enhancing areas (Figure 1A and 1B).

**Figure 2:**
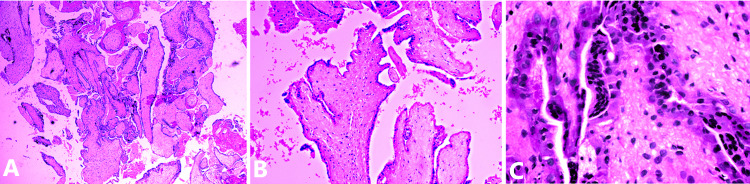
Subcutaneous ependymoma showing the characteristic papillary architecture of the neoplasm. (A) MPE showing a prominent dense slender micropapillary architecture of the neoplasm (H&E ×4). (B and C) - MPE showing papillary structures lined by flattened to cuboidal and elongated tumor cells. The papillary cores are fibrovascular with hyalinization; (B) H&E ×10; (C) H&E ×40.

Wide local excision was conducted on December 21, 2020. Gross examination showed a globular partly solid and cystic mass measuring 14 × 11.5 × 7 cm^3^. The cut surface was solid with cystic hemorrhagic areas. Sections taken from the solid areas showed an ill circumscribed neoplasm with a predominant papillary architecture. The papillae were lined by low columnar to cuboidal epithelial cells. The papillary cores showed a myxohyaline mucinous matrix (Figure 2A-2C).

**Figure 3:**
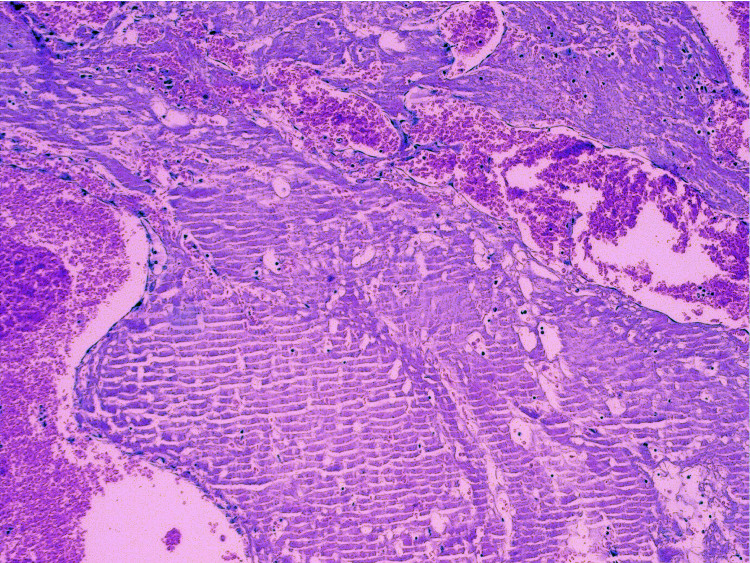
Areas of edema and congestion showing large markedly splayed out papillae with marked myxohyaline degeneration of the papillary core (H&E ×40).

Sections studied from the hemorrhagic necrotic areas showed very large splayed out markedly edematous and necrotic papillae (Figure 3).

**Figure 4:**
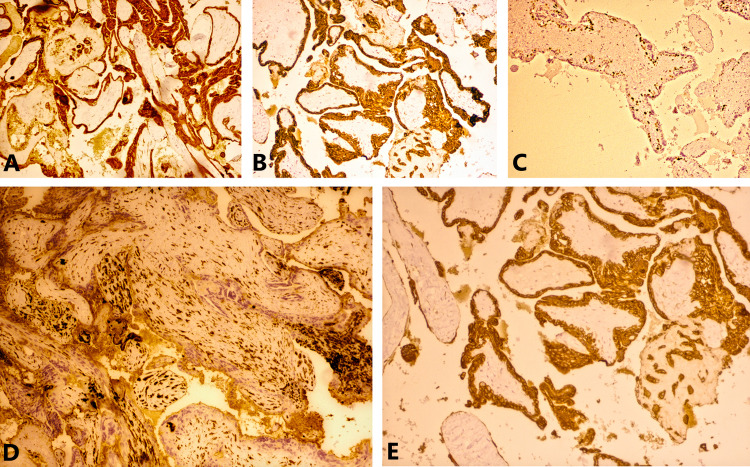
Ependymal cells show strong cytoplasmic positivity for GFAP (A), PAN-CK (B), vimentin (C), S-100 (D), and a high Ki-67 proliferation index of 40–50% (E) (×20). GFAP: glial fibrillary acidic protein, PAN CK: pan-cytokeratin.

On immunohistochemistry (IHC; Table 1), the epithelium lining the papillae were positive for glial fibrillary acidic protein (GFAP; rabbit mono, S-100, cytokeratin, and vimentin (Figure 4A-AD)). Ki-67 proliferation index was around 40-50% (Figure 4E). Further, the epithelium was negative for ER, PR, WT-1, Pax-8, carcinoembryonic antigen (CEA) which ruled out a subcutaneous Mullerian type-ciliated cyst. GFAP and S-100 positivity and CD68 negativity ruled out a subcutaneous metaplastic synovial cyst.

**Table 1:** Details of antibodies and relevant immunohistochemistry protocols used in the study. Sections were deparaffinized and rehydrated. Epitope retrieval was performed in citrate buffer (pH 6) using a multi-epitope retrieval system (Pathnsitu Biotechnologies, Livermore, CA) for five minutes at 120 °C with cooling for 10 minutes before immunostaining. All tissues were then exposed to 3% hydrogen peroxide for five minutes, a primary antibody for 25 minutes, polyexcel target binder for 10 minutes, polyexcel HRP for 10 minutes, diaminobenzidine as chromogen for five minutes, and hematoxylin as a counterstain for one minute. These incubations were performed at room temperature; between incubations, sections were washed with tris-buffered saline buffer.

Name of antibody	Source	Clone	Dilution	Name of supplier
GFAP	Rabbit monoclonal	EP13	Ready to use	Pathnsitu, Livermore, CA
S-100	Rabbit monoclonal	EP32	Ready to use	Pathnsitu, Livermore, CA
PAN-CK	Mouse monoclonal	AE1/AE3	Ready to use	Pathnsitu, Livermore, CA
Vimentin	Mouse monoclonal	V9	Ready to use	Pathnsitu, Livermore, CA
Ki-67	Mouse monoclonal	MIB-1	Ready to use	Pathnsitu, Livermore, CA
ER	Rabbit monoclonal	EP1	Ready to use	Pathnsitu, Livermore, CA
PR	Rabbit monoclonal	EP2	Ready to use	Pathnsitu, Livermore, CA
PAX-8	Rabbit monoclonal	EP33	Ready to use	Pathnsitu, Livermore, CA
WT-1	Rabbit monoclonal	EP122	Ready to use	Pathnsitu, Livermore, CA
CEA	Mouse monoclonal	COL-1	Ready to use	Pathnsitu, Livermore, CA
CD68	Mouse monoclonal	KP1	Ready to use	Pathnsitu, Livermore, CA

**Figure 5:**
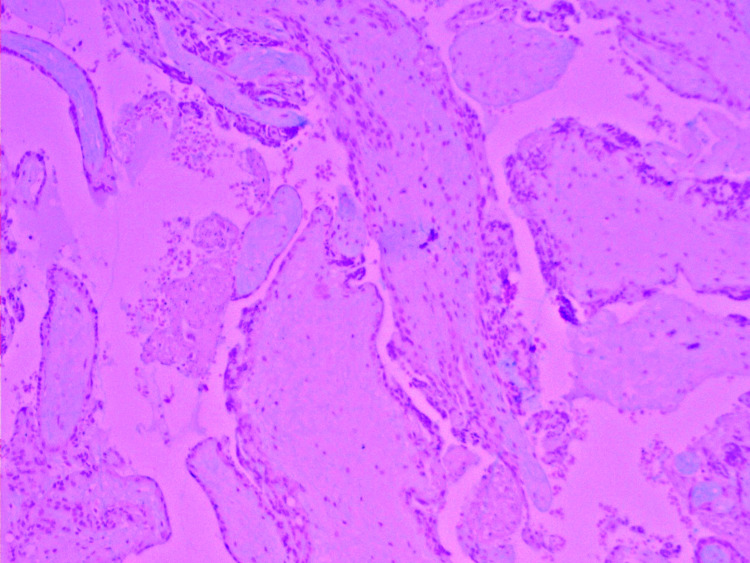
Alcian blue staining highlighting mucinous matrix within the papillary cores of myxopapillary ependymoma (×20).

Alcian blue staining highlighted a mucinous matrix within the papillary cores (Figure 5).

Based on the above findings, a diagnosis of subcutaneous scarococcygeal MPE was made. Close monitoring has been maintained, but to date, there have been no signs of disease recurrence.

## Discussion

History

Mallory was the first to describe an extradural MPE in 1902. Since then, over 50 cases have been identified in the posterior sacral or subcutaneous region [1].

Epidemiology

The average age of MPE onset is around 36 years [3]. The incidence of MPE in males is 0.08 per 100,000 people annually, while in females the estimated rate is 0.05 per 100,000 people per year [10]. Ependymomas account for 60% of all glial spinal cord tumors, and for 90% of the main tumors found in filum terminale and cauda equina. At this caudal location, most ependymomas (as high as 80%) belong to the myxopapillary subtype [11]. Few case reports are available on extraneural metastasizing CNS tumors. The largest by Hoffman et al. included 282 patients, of which approximately 60% were adults and 40% were children. Glioblastoma was the most prevalent metastasizing cancer in adults, followed by meningeal tumors, and medulloblastomas. In these age groups, ependymomas account for nearly 5% of all metastasizing tumors [4,12].

Origin

Spinal ependymomas arise from ependymal cells that line the central canal or its vestiges such as the ventriculus terminalis (“fifth ventricle”) in the conus medullaris or the filum terminale [13]. The coccygeal medullary vestige, a small cavity lined by ependymal cells [13] frequently found in the caudal portion of the neural tube, and subcutaneous ependymal rests are thought to be the origins of most primary sacrococcygeal MPEs. A dimple on the skin surface over the coccyx tip marks the subcutaneous location of the coccygeal medullary vestige [14]. This vestige is found normally in most infants and children (up to approximately 17 years of age) and can give rise to a neoplastic transformation, but such lesions usually regress or involute [14]. Alternatively, MPE may occur outside the spine, especially in the soft tissue situated on the pelvic retrorectal (presacral) space or the posterior side of the sacrum [15].

Sites

**Figure 6:**
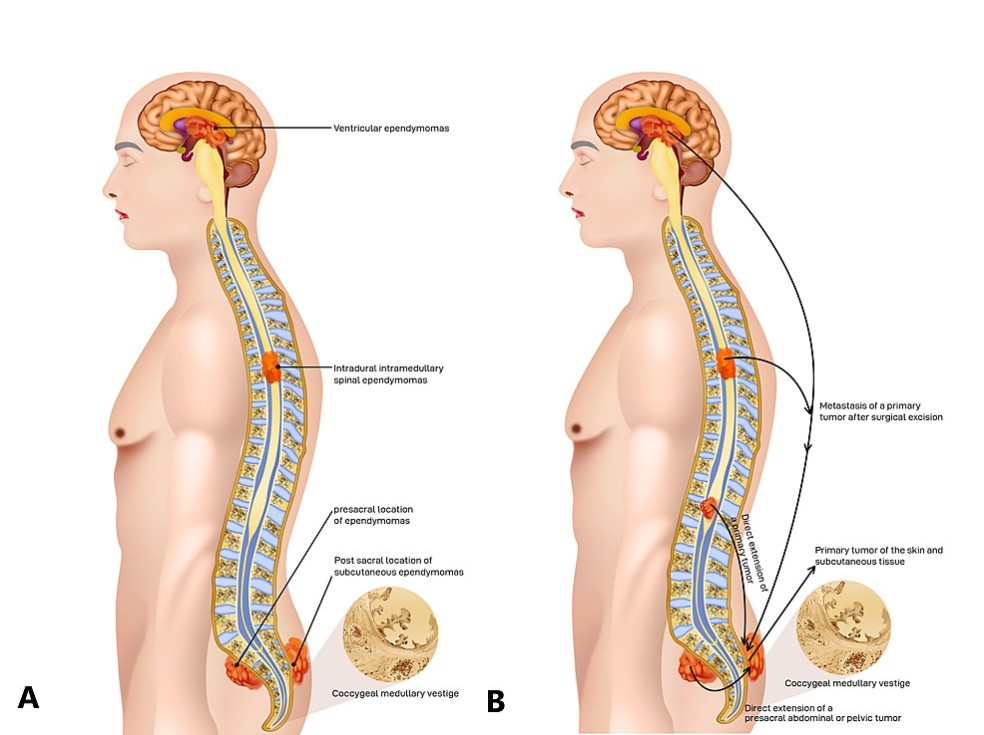
Common locations of spinal and extraspinal ependymomas. (A) Schematic image showing common locations of spinal and extraspinal ependymomas. (B) Schematic image showing subcutaneous ependymoma resulting from primary ependymal cell rests and secondary loco-regional spread of MPEs arising from other sites.

As mentioned, MPEs mainly affect the caudal spinal cord (cauda equina and conus medullaris). Extraspinal (presacral or postsacral) ependymomas are thought to arise from heterotopic ependymal cell rests such as those found in the coccygeal region (Figure 6A) [13]. Postsacral ependymomas usually present as subcutaneous tissue tumors primary to the sacrococcygeal skin with no discernible relation to the filum terminale or spinal cord [16]. Postsacral ependymomas can also form near the gluteal cleft and can be mistakenly diagnosed as a pilonidal disease [2]. Pilonidal sinus, epidermal inclusion cyst, meningocele, lipoma, sacrococcygeal teratoma, and neurogenic tumors are all differential diagnoses for sacrococcygeal lesions [8].

Postsacral extraspinal ependymomas can also arise following surgical excision, from distant metastases, or from the direct expansion of a primary CNS tumor [17], such as the immediate extension of a primary ependymoma of the filum terminale, cauda equina, or lower spinal cord to the sacrococcygeal soft tissue (Figure 6B). Distant sources include vaginal, primary presacral, and abdominal tumors [18]. Ectopic ependymomas have also been found in the lung, mediastinum [16], ovary [19], and uterosacral ligament [16].

Age of presentation

Subcutaneous sacrococcygeal MPEs develop gradually and hence are usually large by clinical presentation. In one series, the average age at presentation was 17 years but ranged widely from 10 months to 47 years [18]. This age range distinguishes subcutaneous sacrococcygeal MPEs from cauda equina MPE, which present between 6 and 82 years. Further, the male-to-female ratio (2.2:18) also differs from cauda equina MPE.

Radiographic findings

**Figure 7:**
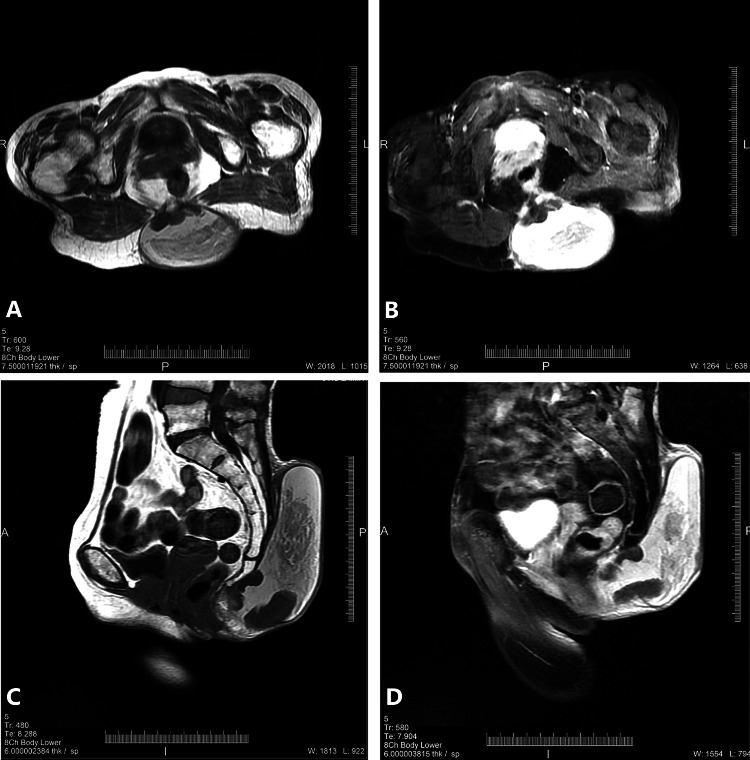
Axial/sagittal T1 and T2 images (plain and post-contrast). (A) and (B) (post-contrast): axial T1 and T2 FAT Sat images - a well-defined thin wall cystic lesion in the subcutaneous plane at the intergluteal cleft region, the intermediate signal intensity of internal solid component with high signal fluid in both T1 and T2WI. No evidence of muscle involvement. (B) and (D) (post-contrast) sagittal T1 and T2WI: a large well-defined thin wall predominantly cystic lesion with an internal solid component at the subcutaneous plane in the sacrococcygeal region, no sacrococcygeal bone involvement.

The imaging features of MPEs are not always unique [20]. On radiographs, subcutaneous MPEs typically appear as broad, well-defined, and encapsulated masses with punctate calcification [20]. Computed tomography (CT) scans often demonstrate a well-defined soft tissue attenuation mass with a possible bony remodeling in the retrosacral area (Figure 7A and 7C) [20]. A mass of soft tissue density is visible on contrast-enhanced CT after intravenous administration of contrast agent (Figure 7B and 7D) [21]. The imaging characteristics of ependymomas developing outside the CNS have not been thoroughly studied, but the MRI and CT manifestations appear similar to those developing within the spinal canal.

MRI also frequently reveals a lobular mass that appears hypo- or isointense to muscle on T1-weighted (T1W) images and hyperintense on T2-weighted (T2W) images, with scattered hypointense fibrovascular septa and covered by a hypointense fibrous capsule. On T1W images, MPEs show homogeneous low signal strength within the mass, while intravenous gadolinium administration results in the appearance of focal nodules at the periphery with streaks of enhancement extending through most of the mass or in stronger homogeneous enhancement. On T2W images, MPEs present a strong signal (with intensity slightly lower than adipose tissue) inside a well-circumscribed mass. The majority of MPEs are circumscribed, but some may have invaded the sacral bone or adjacent soft tissue by a presentation [20].

This heterogeneous contrast enhancement on T2W images is due to the association of the perivascular mucinous stroma with fibrous tissue. The mass frequently also has a peripheral rim of low signal due to local deposition of hemosiderin. In addition, necrosis and hemorrhagic areas may appear, and such lesions are more likely to be cancerous [20]. Subcutaneous MPEs along with neurogenic tumors, sacrococcygeal teratomas, and pilonidal cysts [18,20] should be included in the differential diagnosis of pericoccygeal masses due to shared imaging features. All patients with these kinds of masses should also receive MRI accompanied by image-guided biopsy. After diagnosis, the rest of the CNS must be scanned to rule out other ependymomas.

Classification

In light of frequent recurrences, MPE has been upgraded to WHO grade 2 as per the WHO classification of tumors of the nervous system (preview of the upcoming 5th edition, 2021). Over 15-35% of MPEs can spread hematogenously to the pleura, lungs, local subcutaneous soft tissues, and bone [22].

Pathogenesis

The ependymal cell rests found in the filum terminale are thought to be the primary source of MPEs. Light microscopic findings reveal that aggregation of myxoid content in perivascular spaces distorts the typical ependymal architecture. Direct release of myxoid material by tumor cells and vascular leakage through endothelial fenestrations have been suggested as potential pathways for deposition [23]. However, ultrastructural evidence suggests that myxoid material deposition could be due to excessive development of basement membrane surrounding tumor cells due to contact of collagenous elements in the filum terminale with local ependymal cells [24]. In tissue culture, basement membrane production was observed in the interphase between substrate collagen and neuroglial elements of the rat spinal cord [25]. Further, a rise in basement membrane content closely associated with collagen in the extracellular space has been observed in MPE organ culture.

Gross structure of MPEs

The pathologic presentations of 32 first subcutaneous sacrococcygeal MPE cases were reviewed by Helwig and Stern [14]. The average tumor diameter was 4 cm and ranged from 1.7 cm to 12 cm. The tumors appeared encapsulated, ovoid, and circumscribed, with a rubbery or firm texture. Soft tumors were identified in a few cases. The cut surface was typically lobulated and gray-white in color. Cystic, mucoid areas, hemorrhagic areas, and (or) yellow foci were noted on occasion. Under light microscopy, the tumors exhibited a complete or partial papillary architecture. Patient age and imaging manifestations can help to confirm the diagnosis, particularly if the lesion contains fat. More than half of subcutaneous sacrococcygeal MPEs also show calcification or ossification [18].

Macroscopic appearance

Macroscopically, cauda equina MPEs appear as enlarged, sausage-shaped tumors with a smooth lobulated surface, ranging in size from 10 cm to more than 40 cm in the largest dimension [23]. Large tumors are associated with the destruction of the sacrum. The majority of tumors in this area are well established and demarcated, with disruption and compression of the nearby cauda equina nerve roots. A lobulated, smooth, grayish presentation with focal zones of new and older hemorrhage may appear in cut segments. Calcification is less common than that in other ependymomas.

Histopathology

The word "myxopapillary" refers to the proclivity of these neoplasms to produce mucin and form papillae as a result of arborizing vasculature, thereby markedly altering the architecture of the filum terminale [26].

On histological sections, MPEs are distinguished by a dense, slender, micropapillary bundle covered with cuboidal to elongated tumor cells organized in papillary structures surrounding hyalinized fibrovascular cores. Cell nuclei are usually rounded, with fragile, transparent (Figure 2) chromatin, and a modest amount of amphophilic cytoplasm. The papillae cores are composed of blood vessels enclosed by variable amounts of aPAS-positive myxohyaline mucinous matrix (Figure 7). The vessel walls are thickened and highly hyalinized. Some tumors contain components that mimic other more common ependymomas [24]. Mitotic activity is minimal to nonexistent with low cytologic atypia [25]. While nerve roots can be enclosed, MPEs typically have clearly demarcated margins. A condensed connective tissue capsule wraps part of the tumor [11].

Immunohistochemistry

Cells are typically immunopositive for vimentin, GFAP, S-100 protein, and immunohistochemical cancer markers, while cytokeratin immunoreactivity is relatively uncommon (Figure 4) [27].

Electron microscopy

Cilia, complex interdigitations, and abundant basement membrane material have been observed under electron microscopy. In addition, microtubule aggregation within rough endoplasmic reticulum complexes is a distinguishing feature of some cases [28]. Occasionally, polar inversion may be found when the microvillus-bearing apical surface of one cell directly touches the flat basal surface of a neighboring cell [17].

Molecular pathology and cytogenetics

There have been only a few reported cases of MPE with distinct genetic and cytogenetic abnormalities. In one such case, chromosome 1p rearrangement was noted. In another, tumor tissue sequencing revealed truncation of CTNNA1, which encodes the cadherin family cytoskeleton-associated protein alpha-catenin. This truncation undermines tumor suppression in epithelial tumors (breast cancer, colorectal cancer, and endometrial carcinoma) [25] and leads to cancer cell metastasis [25,29]. A subcutaneous sacrococcygeal MPE with dicentric chromosomes and chromosome 11q deletions has also been reported. In addition, a cytogenetics study identified a t(12;22) EWS translocation and EWS/ATF1 gene fusion [19]. These analyses could provide clues to the molecular pathogenesis of MPE.

Molecular characterization

Molecular characterization has revealed several MPE subtypes, and so may facilitate improved clinical management. Histopathologic criteria have been used to classify ependymomas into grade I MPE (subependymoma), grade II or III ependymoma, RELA fusion-positive grade II ependymoma, and grade III anaplastic ependymoma [5]. A recently developed molecular categorization system for ependymomas has provided a potential method to predict tumor aggressiveness and efficacious therapeutic targets. Pajtler et al. used DNA methylation profiling to divide 500 ependymal tumors into nine molecular subsets [30]. Both spinal ependymomas and spinal MPEs demonstrated comparatively greater consistency with histopathological subtype grades II and I, while the molecular profiles of supratentorial ependymomas and posterior fossa ependymomas frequently did not correspond to histopathologic grade, underscoring the potential clinical utility of this molecular classification system.

The highest frequency of genomic variation among ependymomas, specifically, depletion of 22q, has been observed in SP-EPN tumors (19 of 21; 90%) [24]. However, 22q loss was observed in only a small proportion of SP-MPEs and was not found exclusively in SP-EPN tumors. Further, molecular characterization is currently limited by the rarity of extradural MPE. This molecular classification approach, however, will almost certainly yield important information for predicting the behavior of such tumors. In light of these preliminary results, molecular profiling is recommended for rare tumors like extradural MPE.

Approach to a Case of Subcutaneous Ependymoma

Pathologists working with sacrococcygeal surgical specimens should be aware that these neoplasms may emerge from ependymal cells remaining within the coccygeal medullary vestige and manifest as subcutaneous masses of varying size. As a result, diagnosing these rare tumors necessitates a high clinical index of skepticism[A12]. Furthermore, when diagnosing these lesions in excision biopsies, fine-needle aspiration cytopathology (FNAC) samples, and core biopsies, a suitable histopathologic diagnostic procedure must be used. The clinical strategy for diagnosis of any sacrococcygeal mass should start with an examination of associated neurological defects and somatic symptoms. Sphincter dysfunction, lower back pain, and fatigue in the lower limbs are all common symptoms of filum terminale and cauda equine tumors. Furthermore, radiologic and clinical evidence of intraspinal contact suggests possible spinal dysraphism, which must be ruled out. If there are no neurological deficits, SE should be added to the list of possible diagnoses.

Differentiating Subcutaneous Ependymoma From Ependymal Cell Rests

The most important consideration when verifying an ependymomatous lesion is to distinguish between neoplastic ependymoma and remaining exuberant ependymal cell rests. Careful consideration of diagnostic factors can help distinguish between these growths (Table 2).

**Table 2:** Criteria for distinguishing subcutaneous myxopapillary ependymal rests from subcutaneous myxopapillary ependymomas

	Subcutaneous myxopapillary ependymal rest	Subcutaneous myxopapillary ependymoma
Epidemiology	Onset usually before one year of age [31]	Onset in older children and young adults (age range from 5 days to 67 years) [31]
Clinical presentation	A postsacral dimple or acrochordon [31]	Larger, more discrete nodule or plaque [31]
Histology	Well-circumscribed, without atypia, mitoses, or infiltrative growth pattern [31]	The possible presence of infiltrative growth pattern, atypia, and mitoses [31]
Treatment	Local excision [31]	Wide excision and close clinical follow-up [31]

Myxopapillary ependymal cells remaining in the subcutaneous tissue are benign neuroglial heterotopias originating from the filum terminale. They may appear as small masses or dimples, so this structure should be considered when diagnosing presacral acrochordons. Neurologic imaging must be conducted before biopsy when a mass appears in the centerline of the sacral region to assess CNS involvement. It is also vital to differentiate between ER and SME based on histology [31]. A myxopapillary structure of ependymal cells within a mucinous stroma, as well as immunoreactivity for vimentin, GFAP, and S-100, is indicative of ER. On the other hand, SME is a low-grade malignant tumor characterized by mitoses, atypia, and penetration of nearby structures that may emerge from ER. Moreover, SMEs can metastasize, necessitating broad excision, and careful clinical monitoring thereafter, while ER can be managed with simple resection [31].

Cytomorphologic Approach to a Case of Subcutaneous Ependymoma

The FNAC of MPE is distinguished by an excess of mucinous material (myxomatous as the name suggests). However, mucinous metastases (including metastatic mucinous adenocarcinoma, MPE, extraskeletal myxoid chondrosarcoma - EMC), bone tumors, and myxomatous primary soft tissue tumors (chordoma, myxoid liposarcoma, and soft tissue myoepithelioma) are linked to the sacrum and parasacral tissues. These lesions constitute a difficult differential diagnosis list due to variable cytomorphologic and histologic characteristics within the type and shared features between types, particularly the defined myxomatous or mucinous history [32,33]. In addition, the background material itself can obscure distinguishing features. However, accurate diagnosis is possible by paying close attention to cell type (Table 3).

**Table 3:** Distinguishing cytomorphologic features of subcutaneous ependymomas from other closer differential diagnoses. EMC: extraskeletal myxoid chondrosarcoma, MPE: myxopapillary ependymoma.

Cytomorphologic feature	MPE	EMC	Metastatic adenocarcinoma	Chordoma
Characteristic tumor-associated stroma.	Round, well-defined, extracellular stromal globules either as isolated or centrally located structures and surrounded by ovoid cells. These structures are characterized by a ring of small ovoid cells surrounding a densely granular/fibrillary and intensely magenta-colored globule of extracellular material. These structures are observed consistently in reported cases and are considered to represent the central core of the rosettes [33].	The myxoid chondroid fragments observed in EMC are not as well defined or rounded as the stromal globules identified in MPE and do not have a fibrillary appearance. Moreover, the myxoid chondroid stromal fragments of EMC frequently entrap cells, rather than being surrounded by cells [32].	Metastatic mucinous adenocarcinomas are characterized by a pale blue background and a homogeneous or mucoid appearance without granularity or a fibrillary appearance [32].	The stromal substance has a more granular or fibrillary appearance [32].
Characteristic tumor and cytomorphologic architecture.	The prominent papillary architecture of the stroma with central capillary cores [33]. Prominent cytoplasmic processes associated with spindle-shaped cells [17].	Chondroid fragments containing neoplastic cells within lacunae. In addition, the presence of chondroblasts with multiple small cytoplasmic vacuoles strongly supports a diagnosis of chondrosarcoma [34].	The papillary structures observed in mucinous adenocarcinoma are composed of large numbers of epithelial cells. In contrast, papillary aggregates with or without fibrovascular cores are observed in both MPE and metastatic mucinous adenocarcinoma of colonic origin. In some cases, tissue is so closely packed that a central core of fibrovascular tissue cannot be identified. The papillae surfaces are covered by densely packed columnar cells, often with a “picket fence” appearance [32]. Metastatic gastric adenocarcinoma is characterized by small signet ring cells with a single vacuole displacing the nucleus to one side. These cells are considerably smaller than the physaliphorous cells characteristic of chordoma [32]. In metastatic colonic mucinous adenocarcinoma occasional goblet cells are recognized and have a columnar appearance with an elongated vacuole. The nucleus is situated basally within the cell [32].	Physaliphorous cells characterized by large intracytoplasmic inclusions of magenta-staining granular to a fibrillary substance (Diff-Quik) or by large empty-appearing vacuoles, both of which occasionally displace the nucleus toward the periphery. These inclusions and vacuoles generally do not indent the nucleus [35,36]. True physaliphorous cells are considerably larger than the goblet cells or signet ring cells observed in mucinous adenocarcinoma. Moreover, physaliphorous cells demonstrate a low nuclear-to-cytoplasmic volume ratio and may appear multinucleated or binucleated [35]. This appearance is not seen in cases of mucinous adenocarcinoma.

Tinctorial qualities of the background extracellular substance and architectural features often allow accurate diagnosis. When cytomorphologic characteristics are considered appropriately, MPE can be identified and other closely related lesions ruled out. The most helpful features for distinguishing neoplasms within the differential diagnosis list are goblet cells, physaliphorous cells, rosette-like structures, round stromal globules, cytoplasmic processes, signet ring cells, and chondroid fragments with lacunae [32]. The presence of spindle cells along with wispy cytoplasmic extensions and circular extracellular stromal globules suggests MPE. Alternatively, physaliphorous cells are suggestive of a neoplasm or chordoma, while chondroid segments with lacunae surrounded by neoplastic cells appear unique to EMC. Mucin-rich adenocarcinoma is strongly suggested by the presence of tiny goblet cells or signet ring cells in a light blue mucinous matrix [32].

Histomorphologic Approaches for Diagnosis of the Current Ependymoma Case

The histomorphologic approach to subcutaneous ependymoma diagnosis requires the elimination of other morphologically similar neoplasms. These include a group of neoplasms with villopapillary structure (Table 4) [37,38], myxohyaline tumor matrix, and intracellular or extracellular mucin pools [39,40] in common with MPEs (Table 5).

**Table 4:** Histomorphological and immunohistochemical differentiation of sacrococcygeal lesions with villopapillary architecture.

Lesions with villopapillary tumor architecture in the sacrococcygeal region	Subcutaneous sacrococcygeal myxopapillary ependymoma	Cutaneous Mullerian ciliated cyst	Cutaneous metaplastic synovial cyst
Embryological origin	Developmental origin from coccygeal medullary vestige.	Ectopic Mullerian remnants during early embryogenesis proliferate into a cyst following hormonal stimulation after puberty. Through eccrine metaplasia, manifested by ER and PR negativity [37].	Previous/recurrent trauma, surgical procedures, cutaneous fragility, and anomalous scarring are few hypothesized causes [38].
Tumor architecture and histomorphology	A prominent papillary architecture with characteristic ependymal rosettes. The papillary cores show mucinous stroma and are lined by low columnar to cuboidal ependymal epithelium.	A prominent papillary architecture with fibrovascular cores. The papillae show lining with Mullerian salpingeal type stratified ciliated low columnar to the cuboidal epithelium [37].	A prominent villiform architecture lined by synovial type epithelium and fibrin. A mixture of epithelioid, fibroblastic, mononuclear, and giant cells can also be seen in the lining [38].
Immunohistochemistry	IHC positive for GFAP, S-100, and rarely PAN-CK. Negative for ER, PR, WT-1, PAX-8, and CEA.	IHC-Positive for ER, PR, WT-1, PAX-8, and PAN-CK. Negative for CEA [37].	IHC-positive for vimentin and CD68. Negative for GFAP, S-100, CK, ER, PR, WT-1, PAX-8, and CE [38].

**Table 5:** Features distinguishing myxopapillary ependymoma from similar tumors. MPE: myxopapillary ependymoma, EMA: epithelial membrane antigen.

	MPE	Chondroma/chondrosarcoma	Metastatic adenocarcinoma	Chordoma	Cauda equina paraganglioma
Gross	Usually enlarged, sausage-shaped tumors with a relatively smooth lobulated surface. Tumors can range in size from 10 cm to more than 45 cm in diameter. Large tumors may lead to loss of the sacrum. The majority of tumors in this location are well established and circumscribed, with compression, and disruption of the nearby cauda equina nerve roots. Cut segments have a smooth, lobulated, grayish appearance with focal zones of new and older hemorrhage. Calcification is less common than in other ependymomas.	Light blue or pearly white in color with focal zones of calcification. Tiny cysts or myxoid changes are possible.	Grossly infiltrative, poorly defined damaging lesions.	The cut surface is gelatinous to the chondroid. Chordomas are expansile lobulated masses that typically invade surrounding soft tissue and pervades the cortex.	These tumors are normally well-defined, lobulated growths inside the sacral canal, and can be difficult to differentiate from MPE.
	A prominent papillary architecture of the neoplasm with a myxoid/mucinous matrix in the papillary core. Characteristic ependymal rosettes.	The presence of typical cartilaginous elements distinguishes most chondromas and low-grade chondrosarcomas from MPE. These tumors also exhibit lacunar spaces with tumor cells scattered throughout.	Papillary structures and mucin pools may be present. Intracellular mucin production can be easily detected by staining. Metastatic carcinoma is associated with severe pleomorphism and other characteristics, such as necrosis, that suggest malignant biological activity.	These lesions are widespread in the cauda equine area and have a distinctive myxoid matrix that is Alcian blue-positive. At least some tumor cells show physaliphorous cytoplasmic vacuolation. Unlike MPE, which takes on a papillary growth pattern, chordoma cells form chords and are rooted in the myxoid matrix.	On histology, these tumors lack the well-arranged papillary structure and mucinous matrix of MPE and have a typical reticulin-enclosed cellular nest pattern.
	Positive for GFAP, S-100. Occasionally cytokeratin positive {39},	Negative for GFAP [39].	Positive for cytokeratin [40]. However, variable cytokeratin expression may also occur in ependymoma, so differentiation should not be based on this criterion alone. EMA cannot be used to distinguish between these two entities.	Typically negative for GFAP but positive for cytokeratins [40].	Immunoexpression of neuronal antigens like neurofilament and synaptophysin differs from ependymomas. In paragangliomas, cytokeratin is often expressed. Paraganglioma sustentacular cells are positive for S-100 protein [40].

Myxopapillary ependymoma, chordoma, adenocarcinoma, and myxoid chondrosarcoma can be distinguished using immunohistochemistry. However, immunoexpression levels of GFAP, cytokeratin (CK), S-100, and epithelial membrane antigen (EMA; Table 6) often differ among ependymoma types [41], but frequent conflicting immunohistochemical features within type and overlap among types preclude reliable differentiation.

**Table 6:** Immunohistochemical features of Myxo papillary ependymoma

	GFAP ^39^	S-100 ^39^	CYTOKERATIN AE1/AE3^39^	CEA ^39^
Myxopapillary ependymoma	+/−	+ (50%)	+/−	−
Extraskeletal chondrosarcoma	−	+	−	−
Metastatic adenocarcinoma	−	−	+	+
Chordoma	+ in few cases	+	+	−
Soft tissue myoepithelioma	Variably positive	+	+	−

Furthermore, when there are many regions of necrosis, multiple tumor samples are required for diagnosis as these will aid in the exclusion of ependymomas resulting from a teratoma background. Teratomas occurring on a history of malignant mixed germ cell tumors, which may also develop in the centerline, should be ruled out with caution. The full evaluation and treatment of such lesions will be aided by thorough tumor screening and immunohistochemical and histomorphological evaluation as well as assays for serum tumor markers such as alpha-fetoprotein (AFP), human chorionic gonadotropin (HCG), and lactate dehydrogenase (LDH).

However, there are a few instances where the growth pattern of MPEs is more solid, resulting in perivascular epithelioid cell aggregates showing cytoplasmic clearing and occasional cribriform glandular spaces. In these lesions, the vascular centers of papillae critical for diagnosis are seen only focally [15,18] or not at all, making the distinction from other malignant tumors difficult. In such cases, clear cell sarcoma should also be considered in the differential diagnoses. Clear cell sarcoma may also present with well-circumscribed masses composed of nests, fascicles, or trabeculae of uniformly fusiform or rounded cells with eosinophilic to clear cytoplasm and separated by delicate fibrous septa. Mitotic activity is also normally undetectable in clear cell sarcomas, which may lead to misdiagnosis as a benign tumor. Judicious use of an IHC panel consisting of GFAP, S-100, HMB-45, and Melan-A can help rule out MPE in such cases.

Clinicopathologic correlation

Spinal MPEs generally show a propensity for local recurrence and sluggish progressive growth. However, intracranial multifocality, local spinal dissemination, and extraneural metastasis have been reported. Metastasis to the bone, liver, regional lymph nodes, and lungs occurs in approximately 20% of subcutaneous sacrococcygeal MPE cases, often 10-20 years after the first appearance [27]. Helwig and Stern reported [14] mitotic figures in exceptional situations that did not associate with sacrococcygeal MPE metastatic activity [14]. As a result, mitotic behavior, cytological characteristics, extensive mucinous deposition, and hyalinization tend to have little effect on prognosis. The average Ki67 labeling index of disseminated spinal MPEs was 2.4%, which does not indicate metastasis. MPE with intracranial metastasis does not exhibit any histological signs of malignancy [28]. In contrast, the average proliferation index of disseminated anaplastic ependymomas was 21% [28].

In a study of 32 subcutaneous sacrococcygeal MPEs, Helwig and Stern found 17% cases of metastasis, with the majority occurring in the lung and inguinal lymph nodes [14]. Metastases have also been reported in the skull vertex and vertebral bodies [13]. According to Lee et al. [1], extrameningeal ependymomas metastasize more often than intrameningeal ependymomas that arise in the cauda equine. The clinical appearance is determined by several factors. Symptoms of a pelvic tumor include a noticeable mass during the rectal or pelvic inspection, bowel or bladder impairment, and in some cases, the involvement of sacral nerve roots leading to symptoms like a failure of ankle jerk and/or diminished rectal sphincter tone [15]. MPEs have been reported to spread throughout the body, particularly to extraspinal location [30].

Differential diagnosis

Extradural MPE should be considered in the differential diagnosis of any patient with a slowly growing soft tissue mass in the pre-or postsacral area. Thus, any lesion, particularly near the spine midline, should warn the clinician of a possible underlying ependymal rest or spinal dysraphism as the etiology for abnormalities within the extradural tissue, particularly in young patients. Neurogenic tumor, sacrococcygeal teratoma, pilonidal cyst, lipoma, teratoma, chordoma, metastatic mucinous carcinoma, myxoid chondrosarcoma, metastatic carcinoid, and myxoid soft tissue tumors are among the differential diagnoses of subcutaneous MPE [1].

Sacrococcygeal teratomas can appear as cystic or solid, but they are most frequently cystic. While a solid teratoma may be misdiagnosed as sacrococcygeal MPE, most sacrococcygeal teratomas are found in subcutaneous sacrococcygeal MPEs [18]. Congenital tumors (dermoids, chondromas, and teratomas), inflammatory disorders (internal fistulas and perirectal abscesses), metastatic tumors, other miscellaneous lesions, neurogenic (neurofibroma), anterior meningoceles, and osseous neoplasms are among the differential diagnoses for pelvic MPE.

Treatment

Surgery, Either En Bloc or Gross Total Resection

Extradural MPE has a propensity for spreading to the lungs, lymph nodes, and liver [3]. As a result, obtaining local control of the main location with either gross total resection (GTR) or en bloc resection is critical. Complete surgical excision is recommended as it results in a 98% five-year survival rate. Nonetheless, there is still a 41% risk of regional recurrence, particularly if the surgical excision is incomplete [22]. Retrospective studies on spinal MPE have found that relapse risk is higher following stereotaxic radiosurgery (STR) than GTR or en bloc resection.

Postoperative Complications

A variety of postoperative complications have been reported across studies. For instance, Chakraborti et al. [8] reported wound infection in 15-20% of cases, fecal incontinence in 14.7%, postoperative constipation in 5.9%, and bladder impairment in 1.8% after sacrococcygeal teratoma resection. Recurrence may occur as a result of non-resection of the whole coccyx, incomplete removal, tumor spillage, or the development of a new primary tumor. Other rare complications include rectoperineal fistula, draining sinus, and wound dehiscence [8]. Even in cases of recurrence, Chakraborti et al. reported no signs of neuropathic bladder or bowel disease.

Role of Radiotherapy

Retrospective evidence indicates that salvage radiotherapy (RT) may slow disease progression in cases of deteriorated capsular integrity following partial MPE resection [3]. Adolescent subjects without previous RT history and individuals receiving only STR showed substantially greater rates of recurrence and advancement of intradural MPE.

Patients who received GTR demonstrated a recurrence rate of approximately 10%, substantially lower than the 19% of patients receiving subtotal or piecemeal resection [3]. Furthermore, in cases of metastatic disease, incomplete adjuvant therapy response, or sustained attachment of the tumor to the coccyx, additional radiotherapy or coccygectomy may still be required [1]. Despite the lack of evidence for a consistent correlation between the radiation dose and tumor growth, most institutes now recommend doses of 40-50 Gy for MPE [3].

Adjuvant RT is being used more often to prevent recurrence after resection. A statistically significant increase in local maintenance and progression-free survival (PFS) was noted in a study of 35 patients receiving RT and surgery compared to patients receiving only surgery [40]. A separate multicenter study conducted on 85 patients [42] reported that high-dose RT post-surgery was the only independent predictor of PFS in patients with primary MPE. To date, the preponderance of evidence indicates that postoperative RT is indeed effective and safe as first-line combination therapy for MPE and should be considered irrespective of surgical resection completeness, particularly among younger patients.

This conclusion, however, is based on a series of retrospective studies conducted on spinal MPE patients rather than extradural MPE patients, and none of the aforementioned studies found a substantial difference in overall survival when adjuvant RT was added [8]. In addition, there is inadequate qualitative evidence for RT efficacy against oligometastatic foci as indicated by greater survival rate or longer relapse-free survival. Therefore, larger-scale prospective studies evaluating the broad therapeutic efficacy of resection with and without adjuvant RT are urgently required.

Role of Chemotherapy

Chemotherapy has been used in only a few MPE cases, including for young children with ependymomas, for treatment of tumors highly resistant to RT [5], and in cases of remote metastasis, so the clinical value of this systemic therapy is uncertain. However, it was found that tyrosine kinase inhibitors slowed MPE progression [3]. It was also reported that the platelet-derived growth factor receptor antibody imatinib maintained disease-free status for 11 months following the failure of temozolomide treatment [9]. Another study found that sorafenib treatment of three metastatic intrathoracic MPE cases detected 20 years after early coccygeal removal resulted in disease stability and satisfactory quality of life for one year before disease progression [3,30]. To ensure that there is no distant or local recurrence, observation, and repeated imaging are recommended. Since these tumors can recur locally or become metastatic, long-term monitoring is required.

## Conclusions

Primary sacrococcygeal MPE is slowly growing and usually treatable, but may recur and spread to the lungs, lymph nodes, or spine. After the formation of metastases, the disease progresses slowly but steadily. Long-term monitoring is important because distant and locoregional recurrence can develop years after removal of the primary tumor. The current case-patient emphasizes the significance of careful case reporting for choosing the best possible treatment plan for patients with unusual or rare diseases. This case report and appended review also add to our body of knowledge on ependymomas by presenting evidence-based treatment and follow-up approaches. Following full or near-complete resection, MPEs have a positive prognosis, with greater than 10 years of survival. Subcutaneous sacrococcygeal MPEs still show substantial rates of recurrence after a long delay and a tendency for remote metastasis.
